# Creating a Pipeline of Talent to Feed the Growth of Neuroscience: Lessons From Ghana

**DOI:** 10.1212/NE9.0000000000200199

**Published:** 2025-02-10

**Authors:** Thomas Amatey Tagoe, Hephzi Angela Tagoe

**Affiliations:** 1 Department of Physiology, University of Ghana, Accra, Ghana;; 2 Biomedical Engineering, Academic City University College, Accra, Ghana; and; 3 G.H.Scientific, Accra, Ghana

## Abstract

The fields of science, technology, engineering, and mathematics (STEM) are crucial for innovation and societal advancement. However, gender disparities persist, particularly in neuroscience. This is despite the work of many organizations and initiatives to build capacity among students, with very little done to raise awareness about potential neuroscience careers. In Ghana, the model of neuroscience experiential days targeted at teenage girls is being used to address this gap and ignite an interest around neuroscience. Since 2017, these camps have combined hands-on activities with career talks, reaching hundreds of participants and fostering a community of young women interested in neuroscience. This article shares insights from these camps, highlighting their structure, impact, and the challenges faced. By sharing our experiences and providing recommendations, we aim to inspire similar initiatives contributing to increased gender diversity in STEM.

## Introduction

The fields of science, technology, engineering, and mathematics (STEM) have long been recognized as pivotal drivers of innovation, economic growth, and societal advancement.^1,2^ One area that has experienced significant growth in both research and practice is that of neuroscience, with recent advancements that have captured the imagination of both academia and the public.^3-5^ However, neuroscience, like other STEM fields, faces a significant gender gap globally, despite the rapid advancement and scientific progress within the field.^6-8^

This gender gap is evidenced by the low percentage of researchers and professionals within neuroscience who are women. This lack of gender diversity is not only a matter of equity and inclusion but also a crucial factor in enhancing the overall quality of scientific research. Although this gender gap is a global phenomenon, it is more pronounced in the Global South where neuroscience is experiencing the most rapid growth.^9,10^ In these areas, increasing the participation of women can lead to a more robust and diverse talent pool, which is essential for addressing complex scientific and technological challenges. In Ghana, where neuroscience is an emerging field, a critical challenge has been the creation of a robust pipeline of talent, particularly among underrepresented groups such as women.^11-13^

To address this issue, G.H.Scientific has been running neuroscience experience days for teenage girls over the past 7 years, with the aim of promoting neuroscience education and gender diversity.^14^ These events combine hands-on experiential activities that allow participants to try out neuroscience research techniques, alongside career talks from accomplished professionals within the various subspecialties. During the period that these NeuroGirl camps have run, we have successfully reached hundreds of participants and fostered a community of young women interested in neuroscience. Our primary goals have been to demystify neuroscience, highlight potential career paths, and empower the next generation of neuroscientists who are women.

In this article, we share insights and lessons gleaned from this initiative over the years and offer practical steps for others who wish to replicate or adapt our model. To this end, we discuss the structure and content of our neuroscience experience days, highlight the opportunities they create for participants, and address the challenges we have encountered along the way. By sharing our experiences, we hope to contribute to the broader effort of increasing gender diversity in STEM and its subspecialties by inspiring similar initiatives globally.

### History of the NeuroGirl Camp

The concept of girl camps, designed to empower and educate girls in various fields, particularly in STEM (science, technology, engineering, and mathematics), has its roots in the broader global movement for gender equality. These fields were not only dominated by men but also often excluded women through the creation of stereotypes and assumptions that limit their participation. These forms of exclusion have contributed to the underrepresentation of women in STEM, reinforcing the perception that these fields are domains reserved for men.^15^

The first initiatives resembling modern girl camps emerged in the United States and Europe in the 1990s, driven by the growing recognition of the importance of fostering interest in STEM among young women. Programs such as Techbridge in the United States, which began in 2000, and Girls Who Code, founded in 2012, were among the early adopters of the camp model.^16,17^ In the United Kingdom, Stemettes, founded in 2013, has become a prominent organization dedicated to inspiring the next generation of women in STEM through activities that teach technical skills and build confidence.^18^

These early camps focused on hands-on learning, mentorship, and community building, providing girls with a supportive environment to explore their interests without the societal pressures and stereotypes that often discourage them from pursuing STEM fields.^12,19^ In Africa, the adoption of girl camps reflects the continent's rapid economic development and the growing recognition of the need to empower women as part of that process. The first girl camps in Africa began to appear in the early 2000s, often organized by nongovernmental organizations, local community groups, and increasingly, by African governments themselves.^11^

One of the pioneering initiatives was the Tech Needs Girls program, launched in Ghana in 2012.^20^ This program was designed to train young girls in coding and technology, addressing the digital divide that disproportionately affects women. Then, there is the *African Gifted Foundation*, which runs STEM summer academies for gifted students across Africa between 2011 and 2013.^21^ These camps had over 200 students attending from 12 African countries, including Nigeria, Cameroon, South Africa, and Sudan. Their activities highlighted the immense potential of students with a passion for STEM and have since morphed into a specialist STEM academy for girls known as the African Science Academy. At the African Science Academy, students complete a 2-year A-level program in 10 months, graduating with A-levels in maths, physics, and further maths.^22^

Another significant initiative was the African Girls Can Code Initiative (AGCCI), launched by the African Union Commission in partnership with UN Women and the International Telecommunication Union in 2018.^23^ AGCCI aimed to equip young African women with digital literacy skills, coding abilities, and leadership training, reflecting a broader continental effort to increase participation of women in the digital economy. Finally, in South Africa, GirlEng was established as part of the Women in Engineering (WomEng) organization, focusing on encouraging girls to pursue careers in engineering.^24^ GirlEng camps provided practical engineering experience, mentorship, and exposure to real-world engineering problems, aiming to demystify the field and inspire a new generation of engineers who are women.

These initiatives, although diverse in their approaches, share common goals: to bridge the gender gap in STEM, to empower girls through education and skills training, and to provide role models and mentorship opportunities that can help sustain their interest and development in these fields.

Similarly, the NeuroGirl Camp initiative, conceived and launched by G.H.Scientific, began in 2017 with the goal of addressing the underrepresentation of women in neuroscience and related STEM fields in Ghana. This initiative emerged from recognition of the critical need to inspire and engage young women in science, particularly in neuroscience, where gender disparities are pronounced globally and even more so in the Global South.

### The Inaugural NeuroGirl Camp

The first NeuroGirl Camp took place in 2017 with funding from the International Brain Research Organization (IBRO) as part of its global brain awareness week initiatives. This marked the beginning of a dedicated effort to create a supportive environment where girls could explore neuroscience, interact with neuroscience professionals (men and women), and gain hands-on experience in the field. The inaugural camp brought together 100 girls from senior high schools across Ghana to the University of Ghana Medical School at Korle Bu (Accra, Ghana). The event was designed as an informal and highly interactive day-long experience featuring a mix of mentoring, practical activities, and open discussions.

The camp began with an introductory session that highlighted the contributions of notable neuroscientists who are women, aiming to inspire participants by showcasing what their futures could hold. This was followed by a speed mentoring session, where the girls had the opportunity to engage in one-on-one conversations with professionals from various subspecialties within neuroscience. In the afternoon, the participants engaged in practical sessions providing them with a taste of real-world scientific inquiry. These activities were carefully designed to be both educational and engaging, allowing the girls to apply theoretical knowledge in a hands-on setting. The day concluded with an open forum, providing a platform for the participants to share their experiences, ask questions, and discuss their aspirations with the mentors. A copy of the program handbook given to each student is given in eAppendix 1.

### The Growth and Impact of NeuroGirl Camps

Since its inception, the NeuroGirl Camp has seen significant growth both in scope and impact, evolving from a single-location event into a nationwide initiative with far-reaching influence. Building on the initial success, the 2019 NeuroGirl Camp marked a pivotal expansion, with the program being held in the capital city of 3 regions in Ghana (Cape Coast, Kumasi, and Accra). This expansion was made possible through partnerships with multiple organizations, including local universities, professional societies, and media houses.

Notably, one of the 2019 events held in Accra was designed to host both boys and girls. This inclusive approach represented a significant evolution in the camp's mission, recognizing the importance of engaging all young minds in neuroscience while still maintaining a strong focus on empowering girls. The unisex camp provided a platform for both sexes to collaborate and learn together, fostering a broader understanding and appreciation of neuroscience across the student population.

The sustained success of these camps has been, in large part, due to the dedicated efforts of G.H.Scientific and its network of professionals who volunteer their time and expertise. In addition, funding from IBRO and the Biochemical Society (through its diversity grant) has played a crucial role in ensuring the continuity and expansion of the NeuroGirl camps, allowing the program to reach more participants and deepen its impact. By continuing to adapt and expand the program, we aim to ensure that the growth of neuroscience in Ghana is fueled by a diverse and talented workforce, with women playing a central role in shaping the future of the field.

## Overview of the NeuroGirl Camp

### Format and Structure

In line with the objective of inspiring young women, each NeuroGirl Camp involves a morning session of small group engagements with neuroscience professionals, followed by an afternoon session of experiential learning. This combined approach is supported by evidence that personal experiences with role models are more likely to influence students' decisions.^25^ In addition, hands-on activities are known to enhance curiosity and increase understanding.^26^ This format has continued to receive positive feedback from participants over the years and as such has been maintained.

### Participant Demographic and Selection Process

The NeuroGirl Camp focuses on a specific demographic of Ghanaians: girls enrolled in high school. These girls were typically aged between 11 and 14 years (junior high school) or 14 and 18 years (senior high school). For each event, we aim to host 60–100 girls. As such, invitations are sent to 10 schools within a 20-mile radius of the venue, and each school selects 10 of their students who teachers believe would benefit most from the experience. This approach empowers the teachers and ensures that all participants are interested and engaged. There are numerous occasions where some schools do not respond to the invitation and others bring along more students than they are allocated. This is usually anticipated and planned for accordingly.

### Career Talks

The field of neuroscience has many subspecialties, meaning that there are multiple career paths to choose from.^6^ For the morning session, we invite a variety of neuroscience professionals to engage in small group discussions with invited students. Over the years, we have had 22 different professionals be a part of our mentoring sessions, with some partaking on multiple occasions (Table 1). Based on the number of professionals available, students are split into 6–10 groups to engage in 20-minute small group conversations with each professional present. Where the number of available professionals cannot meet the demand, we have had postgraduate students support the mentoring sessions.

### Hands-on Activities

During the afternoon sessions, a selection of classical and modern neuroscience practical demonstrations is led by postgraduate students and volunteers (Table 2). Students observe some and actively participate in others. The aim of this session is to introduce them to some common neuroscience techniques with the hope of increasing excitement for the future. Based on the choice of activities, 4 to 10 stations are set up and students either rotate through them freely or in groups. Each station is designed to demystify complex scientific concepts, making them accessible and exciting for the students. Students are encouraged to ask questions and take notes to feedback during the debrief at the end of the day.

### Debriefing

At the end of the day, students gather to answer questions, share their experiences, and receive souvenirs as prizes for their efforts. During this period, we also collect feedback, which forms part of our monitoring and evaluation process.

## Opportunities to Contribute Toward the Growth of Neuroscience

Engaging the girls with neuroscience at an early age challenges and reshapes traditional gender norms that often deter young women from pursuing careers in STEM.^12,19^ Therefore, the NeuroGirl camps serve as stepping stones in building a pipeline of talent that feed into both local and global neuroscience training programs at more advanced stages of the academic journey. This creates several opportunities, the most significant of which have been discussed below.

### Creating an Interest in Neuroscience

First, the camps introduce participants to the broad spectrum of career opportunities within neuroscience. Through discussions, presentations, and interactive sessions, the girls learn about various specializations and the practicality of what the specialization entails. They also learn how these fields contribute to understanding the brain and treating neurologic conditions. This exposure is crucial for helping them make informed decisions about their future studies and career paths, ensuring that they are aware of the diverse roles they can pursue within the field of neuroscience. Beyond this, the early exposure and practical hands-on activities enable these girls to experience the excitement of discovery firsthand. This experiential learning is vital for cementing their interest in neuroscience, making it more likely that they will pursue further studies and careers in this field.^26^

### Community Building

A key component of our 1-day neuroscience camps is the focus on career development and mentorship, which plays a critical role in shaping the future trajectories of the participating girls. By connecting these young minds with established neuroscience professionals, we demystify the profession by providing the girls with tangible examples of success, reinforcing the belief that they too can achieve similar heights.^19,25^ Furthermore, the network of peers that the girls build during these camps creates a supportive community that can continue to provide encouragement and opportunities as they progress in their education and careers. Based on the theories of legitimate peripheral participation^27^ and identity formation,^28^ it is expected that our program alumni will gradually increase their involvement in neuroscience activities and engage with the larger neuroscience community both nationally (e.g., the Ghana Neuroscience Society) and globally. We have started to witness evidence of this because some alumni have taken part in the International Brain Bee competition, returned as volunteers, or joined the Ghana Neuroscience Society.

### Exposure to Technical Skills

Practical, hands-on skills are the other key focus of the camps. Neuroscience is a field that relies heavily on practical expertise, from conducting laboratory experiments to analyzing data. The camps provide the girls with opportunities to engage directly with the tools and techniques used in neuroscience research. Whether they are using microscopes to examine neural tissues or setting up animal behavioral experiments, these hands-on experiences give them an introductory reference point and a personal experience. This deepens their practical knowledge and interest in neuroscience, giving them the confidence needed to further explore these essential skills needed to excel in future academic and research endeavors.

### Confidence to Pursue Neuroscience

Confidence is a foundational soft skill that our camps also aim to cultivate. Many of the girls who attend these camps may initially perceive neuroscience as a daunting or inaccessible field. Through guided activities, mentorship, freedom to ask questions, and feedback sessions, we create a supportive environment where they can explore complex concepts. As they engage in experiments, dissect brains, and interpret animal behavior, they gain confidence in their abilities to understand scientific principles and, most importantly, to ask questions when they do not (Table 1).

**Table 1:** Number of Student and Professional Participants (Men/Boys [M] and Women/Girls [W]) Over the Years

	2017 M:W	2018 M:W	2019^a^ M:W	2022 M:W	2023^b^ M:W	2024^a^ M:W
Student participants	0:113	0:112	46:160	0:96	360:610	112:218
Postgraduate student volunteers	2:4	3:22	12:18	4:3	13:17	28:32
Neuropharmacologist	1:2	1:1	3:4	0:1	1:0	—
Neurophysiologist	1:0	1:0	3:2	1:0	2:1	1:0
Speech and language therapist	—	—	—	0:1	—	0:1
Psychiatrist	0:1	—	0:2	1:0	—	—
Clinical psychologist	0:1	—	—	—	—	0:1
Neurologist	0:2	—	0:1	0:1	—	—
Physiotherapist	—	—	—	0:1	—	0:1
Neuroanatomist	0:1	1:1	2:1	—	0:1	0:1
Other professions	0:1	0:6	—	—	—	—

a Multiple events were run in these years.

b Event was run as part of a larger science festival these years.

### Collaborative Work Ethic

In addition to confidence, the camps focus on developing other essential soft skills, such as communication, teamwork, and critical thinking. For example, participants work in groups when visiting the various stations and share their experiences in the form of feedback. This experience helps them to practice articulating their ideas and collaborating effectively with others. By doing so, they learn how to express their thoughts clearly, listen to different perspectives, and work together toward a common goal. These skills are invaluable in any professional or academic setting (Table 2).

**Table 2:** Overview of Practical Activities Conducted Over the Years

Activity	Year					
	2017	2018	2019	2022	2023	2024
DNA extraction		x	x		x	x
Molecular modeling		x	x		x	x
Animal behavioral tests	x	x			x	x
BYB spikerbox	x		x	x	x	x
BYB brain claw	x		x	x	x	x
Sensory reflexes	x				x	x
Microscopy	x	x	x		x	x
Neuroanatomy dissections		x	x	x	x	x
Neurologic examination		x	x		x	x
Reaction time tests		x			x	x

## Unmet Needs and Challenges

The success of the NeuroGirl camps in Ghana underscores the potential of such initiatives to inspire and empower young women to pursue careers in neuroscience and other STEM fields. However, the growth and impact of these camps have also highlighted significant challenges that must be addressed to ensure their sustainability and scalability. These challenges are multifaceted, some of which are addressed in the following sections.

### Logistical and Resource Constraints

One of the most persistent challenges faced by the NeuroGirl camps has been logistical and resource related. These constraints manifest in various ways, from securing appropriate venues to managing budgets that are often tight and reliant on external funding. For instance, the reports from the 2017 and 2022 camps reveal recurring issues, such as delays due to inadequate preparation of venues, food shortages, and technical difficulties with equipment. These problems not only disrupt the flow of events but also diminish the overall experience for participants and mentors alike.

### Stereotype Threat and Bias

Beyond logistical issues, the NeuroGirl camps have had to contend with the deep-seated societal challenges of stereotype threat and bias, which continue to undermine the confidence and aspirations of young women in STEM fields. Stereotype threat—the fear of confirming negative stereotypes about one's social group—remains a significant barrier to the full participation of girls in neuroscience and other technical disciplines. This threat is compounded by the lack of visible role models and the ongoing underrepresentation of women in these fields, which reinforces the perception that STEM is inherently dominated by men.

Although the value of single-gender initiatives is evident, the world of work is typically mixed gender and the social dynamics that accompany mixed-gender interactions cannot be ignored.^29^ Therefore, initiatives that seek to empower girls and prepare them for the future should provide experiences that build the confidence needed to take risks and create supporting networks.^30^ Training should also be provided to help them develop transferable skills, build reputation, and identify opportunities early on in their academic journey. These have been shown to significantly contribute toward successful science careers.^31^ Indeed, initiatives such as Women in STEM (WiSTEM) in Ghana, World Women in Neuroscience (WWN), and The ALBA Network champion these same practices for neuroscientists who are women.

### Sustainability and Scalability

Although the NeuroGirl camps have demonstrated significant impact, their sustainability and scalability remain critical challenges. The current model, heavily dependent on external funding and volunteer support, raises questions about the long-term viability. To overcome this challenge, we leverage collaborations with institutions and professional bodies to provide essential resources and expertise, enabling the camps to offer high-quality experiences despite limited budgets. Expanding these partnerships and exploring new collaborations could provide the necessary financial and logistical support to scale the camps.

## Recommendations When Replicating the NeuroGirl Camp Model

The success of the NeuroGirl Camp in Ghana provides valuable insights for organizations and institutions looking to replicate this model in other parts of the world. To maximize the impact of similar initiatives, careful consideration must be given to the structure, content, and inclusivity of the events. Below are practical recommendations based on the experiences, lessons learned, and challenges encountered during the NeuroGirl camps.

### Practical Tips for Organizing Similar Events

#### Early and Thorough Planning

This allows ample time for securing venues, contacting potential speakers and mentors, and organizing logistics. It also allows time to put in place contingency plans such as backup venues, additional supplies, and technical support to handle unexpected issues on the day of the event. Most importantly, it allows the schools where these students attend to fulfill their own administrative processes in releasing the students to attend the event.

#### Securing Reliable Funding and Partnerships

Sustainable funding, ideally from multiple sources is essential for sustainability. A large multiyear grant coupled with sponsorships of any kind and fundraising activities will be the best for growth and continuity. Partnerships with local universities, professional societies, and media outlets not only provide additional resources and expertise but also help to mitigate resource constraints. These collaborations can also offer cost-sharing opportunities, reducing the financial burden on the organizing body.

#### Volunteer Recruitment and Training

Our access to volunteers meant reduced human resource costs while at the same time providing experiential learning opportunities for the volunteers themselves. It is important to recruit volunteers who are passionate about STEM and have a desire to grow. This makes it easier for them to be assigned additional roles if the need arises. In our experience, comprehensive training to adequately prepare volunteers and clear communication channels increases the likelihood of running a smooth event.

### Measuring Outputs, Outcomes, and Impact

It is always a good idea to put in place trackable measures of success. For us, some of these measures have included repeat participation from schools; repeat funding from the same organizations; and feedback forms completed by students, volunteers, and teachers. We place immense value on feedback from teachers who have accompanied their students to attend multiple iterations over the years. Over the years, we have been unable to maintain a comprehensive database of all student participants, which would have allowed us to follow up on each student's academic progression on a yearly basis. Such a database would have allowed us to measure long-term impact as we follow up with students once they are established in the professional careers. However, because some of our volunteers are faculty at universities across the country, we receive 3 to 5 reports every year of alumni who are now in medical school or studying related courses. Based on this, we recommend organizers put in place a customized database akin to customer relationship management software. This will allow structured longitudinal tracking of all student participants, mentors, and volunteers.

### Recommendations for Career Talks and Activities

#### Diverse and Inspiring Speakers

Career talks are a cornerstone of the NeuroGirl Camp experience. When selecting speakers, we prioritize diversity in terms of professional backgrounds, career paths, and personal experiences. Given the challenge of stereotype threat, it is crucial that these speakers include successful women who can serve as role models. Accessibility is also important, and we encourage speakers to share their personal journeys, including both their challenges and achievements as well as personal details such as salary and how they strive for a work-life balance. All this gives the participants a realistic and relatable perspective that can help counteract the effects of stereotype threat.

#### Interactive and Hands-On Activities

The practical sessions should be designed to be both educational and engaging. Activities that allow participants to conduct real experiments, such as DNA extraction or behavioral studies with model organisms, are particularly effective. However, it is important to ensure that these activities are not overly complex because time and logistical constraints can hinder their execution. Prioritize activities that are feasible within the available resources, and consider scaling up only when the necessary support is secured.

#### Balancing Theory With Practice

Although hands-on activities are crucial, it is equally important to provide theoretical context that explains the relevance of the experiments. We often have short, focused presentations on the scientific principles behind the activities to help participants make connections between theory and practice. This balanced approach ensures that participants not only enjoy the activities but also gain some basic understanding of the science involved; this is essential for sustaining long-term interest in the field.

### Strategies for Engagement and Inclusivity

#### Creating a Safe and Supportive Environment

The NeuroGirl camps have shown that girls are more likely to engage actively when they feel safe and supported. Single-gendered settings are one way to achieve this, although cultural contexts should be considered. Alternative approaches to achieve the same outcomes may include laying ground rules at the start of the day, providing mentorship that addresses gender-specific challenges, and team-building activities between boys and girls to foster group work that encourages collaboration. Ensuring that girls are given leadership roles within these mixed teams will also empower them.

#### Tailoring Content to the Audience

Understanding the background and interests of the participants is key to designing an engaging program. We had different activities tailored for the 2 target age groups (11–14 years and 14–18 years); this was informed by surveys and feedback from previous events that provided insights into what topics and activities resonate most with the target audience. Feedback from teachers was also helpful in this regard. Tailoring content to match the participants' educational level, cultural context, and career aspirations will make the event more relevant and impactful because the content will be more relatable and accessible.

#### Ongoing Engagement and Mentorship

The impact of the NeuroGirl camps has been amplified by the follow-up mentorship programs. Given the challenge of sustaining interest beyond the initial camp experience, providing opportunities for continued engagement through alumni networks, online communities, or regular meetups is crucial. Mentorship programs that connect participants with professionals in their fields of interest offer valuable guidance and support as they navigate their educational and career paths.

#### Inclusivity in Participation

Efforts should be made to ensure that the camp is accessible to students from diverse backgrounds, including those from underrepresented communities. This may involve arranging transportation, providing refreshments, or offering the program in multiple locations to reach a wider audience. The 2019 expansion into 3 regions of Ghana is a model for how geographical inclusivity can be achieved. Ensuring that these events remain free or low cost, while securing sustained funding, will help to maintain accessibility for all participants, regardless of their socioeconomic background.

## Conclusion

The NeuroGirl Camp initiative has demonstrated significant potential in empowering a new generation of girls with an interest in neuroscience and by doing so, bridging the gender gap. By providing teenage girls with hands-on experiences and exposure to career opportunities in neuroscience, the camps have not only sparked interest but also built a supportive community of young women aspiring to enter STEM fields. The insights gained from these camps underscore the importance of targeted educational programs in fostering diversity and inclusion in STEM. Replicating the NeuroGirl Camp model requires careful attention to planning, content, and inclusivity, with a clear strategy to address the unmet needs and challenges identified through previous experiences. Moving forward, it is essential to address the challenges identified, such as resource limitations and societal biases, to ensure the sustainability and expansion of such initiatives. By sharing our experiences and outcomes, we hope to inspire similar efforts globally, contributing to a more diverse and inclusive scientific community.

## Glossary

AGCCI: African Girls Can Code Initiative
IBRO: International Brain Research Organisation
STEM: Science, Technology, Engineering, and Mathematics
